# Determination of Microcystins in Fish Tissue by ELISA and MALDI-TOF MS Using a Highly Specific Single Domain Antibody

**DOI:** 10.3390/toxins15020084

**Published:** 2023-01-17

**Authors:** Natalia Badagian, Macarena Pírez Schirmer, Andrés Pérez Parada, Gualberto Gonzalez-Sapienza, Beatriz M. Brena

**Affiliations:** 1 Biochemistry Area, Department of Biosciences, Faculty of Chemistry, Universidad de la República, Av. Gral. Flores 2124, Montevideo 11800, Uruguay; 2 Immunology Area, Department of Biosciences, Faculty of Chemistry, Universidad de la República, Av A. Navarro 3051, Montevideo 11600, Uruguay; 3 Technological Development Department, Centro Universitario Regional del Este, Universidad de la República, Ruta 9, Rocha 27000, Uruguay

**Keywords:** microcystins, ELISA, MALDI-TOF, fish, nanobody

## Abstract

The development of simple, reliable, and cost-effective methods is critically important to study the spatial and temporal variation of microcystins (MCs) in the food chain. Nanobodies (Nbs), antigen binding fragments from camelid antibodies, present valuable features for analytical applications. Their small antigen binding site offers a focused recognition of small analytes, reducing spurious cross-reactivity and matrix effects. A high affinity and broad cross-reactivity anti-MCs-Nb, from a llama antibody library, was validated in enzyme linked immunosorbent assay (ELISA), and bound to magnetic particles with an internal standard for pre-concentration in quantitative-matrix-assisted laser desorption ionization-time of flight mass spectrometry (Nb-QMALDI MS). Both methods are easy and fast; ELISA provides a global result, while Nb-QMALDI MS allows for the quantification of individual congeners and showed excellent performance in the fish muscle extracts. The ELISA assay range was 1.8–29 ng/g and for Nb-QMALDI, it was 0.29–29 ng/g fish ww. Fifty-five fish from a MC-containing dam were analyzed by both methods. The correlation ELISA/sum of the MC congeners by Nb-QMALDI-MS was very high (r Spearman = 0.9645, *p* < 0.0001). Using ROC curves, ELISA cut-off limits were defined to accurately predict the sum of MCs by Nb-QMALDI-MS (100% sensitivity; ≥89% specificity). Both methods were shown to be simple and efficient for screening MCs in fish muscle to prioritize samples for confirmatory methods.

## 1. Introduction

Cyanobacterial blooms are common in large eutrophic lakes around the world as well as in several estuaries and coastal marine waters [1,2,3,4,5,6]. These blooms have been associated with animal and human poisonings, long before their toxins were characterized in detail [7,8,9]. Microcystins (MCs) are the most frequently reported freshwater cyanobacterial toxins worldwide [10]. In addition to being highly toxic, MCs can reach very high concentrations, up to 10–20 µg/L in pelagic waters and 20–124 mg/L in scums [11], so they are among the most commonly cyanotoxins related to poisonings across all continents [10]. They comprise a family of hepatotoxic cyclic heptapeptides with close to 300 chemical variants with the following general structure: cyclo-(DAla1-X2-D-MeAsp3-Z4-Adda5-D-Glu6-MDha7) [12,13]. They contain a characteristic amino acid, ADDA (2S,3S,4E,6E,8S,9S)3-amino-9-methoxy-2,6,8-trimethyl-10-phenyldeca-4,6-dienoic acid, and two variable L residues (X and Z) at positions 2 and 4. Other multiple variants arise from modifications and substitutions in several amino acids. The microcystin containing leucine and arginine in positions 2 and 4 (MC-LR) is the most representative member of the family [14] and is probably the most toxic, with a 50% lethal dose value (LD50) of 50 µg/kg via intraperitoneal injection (*i.p*.) in mouse [11].

Microcystins bind covalently and inhibit several protein phosphatases, promoting a series of effects such as the disruption of the cytoskeleton, oxidative stress, and ultimately cellular apoptosis and necrosis [15,16,17,18]. High doses via *i.p*. can be fatal due to sinusoidal capillary damage and hepatic hemorrhage [7,19]. Exposure through the water of renal dialysis led to the first documented case of human lethality caused by MCs as the major factor of death [7,20]. Chronic toxicity is also a problem as long-term repeated exposure induces hepatic hypertrophy and shows tumor promoting activity [21]. The Working Group on the Evaluation of Carcinogenic Risks to Humans (IARC) classified MC-LR as possibly carcinogenic to humans, Group 2B [22]. Other possible chronic effects include the induction of reproductive toxicity [23], chronic kidney disease [24] as well as colorectal chronic inflammation and fibrosis [25].

Microcystin-LR was the first cyanotoxin for which the World Health Organization (WHO) defined in 1998, a provisional guidance value in drinking water (1 µg/L) based in a tolerable daily intake (TDI) for chronic exposure of 0.04 µg/kg body weight per day [26]. This TDI and the corresponding lifetime drinking water values have been maintained up to the present. In 2020, two new provisional guidance values for MCs were added: a short-term value in drinking water (12 µg/L) and a recreational water value of 24 µg/L [27]. These guidance values were derived for MC-LR, but the WHO recommended using the sum of all MCs, assuming the worst-case scenario in which all the congeners have a similar toxicity to MC-LR.

Exposure to MCs can also occur through the consumption of contaminated food or nutritional supplements [28,29]. MCs can accumulate in fish and other aquatic organisms and numerous reports have revealed that this is a global problem [30,31,32,33]. The highest accumulation occurs in shellfish and in fish with viscera, but even in fish muscle, several articles have reported MC concentrations above the short-term TDI [28]. Due to the lack of adequate toxicological data, no widely accepted guideline values are currently available. Moreover, the reliability of the analytical methodology is critical for the characterization of the occurrence of MCs in fish [30], so the problem is not adequately characterized.

The determination of MCs in fish tissue extracts is complex due to multiple issues such as matrix effects, the need for laborious sample pre-concentration and clean up procedures as well as the occurrence of multiple chemical variants together with degradation/detoxification products. An additional matter is that in tissues, MCs exist in both the free form and bound to proteins, and it is not clear whether the protein-bound toxins are released to a relevant extent in the gut [28,30]. In practice, the focus has been on free MCs, and most extraction methods involve a high percentage of organic solvents [34,35] in which proteins precipitate, so the tissue extracts are expected to contain free MCs together with other soluble metabolites.

Liquid chromatography coupled to tandem mass spectrometry, especially to a triple quadrupole analyzer (LC-MS/MS) is a highly reliable method for the identification and quantification of MCs, even in complex matrices [34,36]. With the use of sample preconcentration and clean up techniques or online procedures, these methods can be highly sensitive and selective for the analytical determination of MCs in the biological samples [37,38,39]. However, with close to 300 variants, the availability of MC standards limits their appropriate optimization of the MS/MS conditions (i.e., collision energies), identification via retention time matching, and quantification. In addition to the high cost of the equipment, the LC-MS/MS technique requires personnel with specialized training and the interpretation of the results may be time consuming [40].

To characterize the spatial and temporal variation of MCs in the food chain, it is critically important to develop simple, fast, and cost-effective methods to effectively manage large numbers of samples. One practical approach is to combine simple and reliable screening tools to prioritize samples that need to be analyzed by confirmatory methods [28]. To this end, immunochemical methods based on antibodies that recognize the multiple variants of the MC family are particularly useful for the analysis. In water, ELISAs are widely accepted as simple, efficient, and reliable screening methods [41,42]. They are highly sensitive with minimal sample preparation and are widely available at reasonable cost. However, the application of ELISA to tissue extracts has been questioned due to the potential matrix effects that may lead to false positive and false negative results and to the cross-reactivity of antibodies with metabolites of lower toxicity than the parent toxin [30,43]. Therefore, the characteristics of the antibody and the assay conditions are of critical importance to the success of ELISA in complex matrices.

Nanobodies (Nbs) are antigen binding fragments derived from camelid antibodies (heavy chain only) that may offer a promising alternative to avoid matrix effects. They are monoclonal in nature, reducing the complexity of the polyclonal antibody binding sites, and have a more focused antigen recognition site involving only three CDRs, as opposed to the six that make up the much larger binding site of conventional monoclonal antibodies. This is particularly relevant for small analytes such as MCs, which only interact with a small fraction of the paratope, and thus leave an important unoccupied region that can be a source of nonspecific cross-reactivity with multiple sample components. Furthermore, Nbs also possess valuable features for analytical applications: ease of selection, production at low cost by fermentation in *E. coli*, good solubility, stability, and simple genetic manipulation [44]. In this work, we used a previously developed Nb (NbA2.3) against MCs, whose crystal structure revealed a strong recognition of the ADDA group (unpublished results). The ELISA developed with this Nb turned out to be highly sensitive and selective for the analysis of the water samples [45]. In a subsequent application, the Nb was used as an immuno-concentration element for the quantitative analysis of MCs captured on magnetic beads, functionalized with NbA2.3, and pre-loaded with a MC-analog internal standard prior to matrix-assisted laser desorption ionization-time of flight mass spectrometry (MALDI-TOF) [46]. This methodology, named Nb-QMALDI MS, is very sensitive and allows for the determination of multiple chemical MC-variants simultaneously by MALDI-TOF MS, without previous chromatographic separation.

In this work, we adapted and validated the Nb-based ELISA and Nb-QMALDI MS methods for the analysis of MCs in fish. Using both methods, we analyzed 55 fish samples taken from a eutrophic dam containing MCs, compared their results, evaluated, and discussed their application for the screening of MCs in fish, with particular emphasis in the risk assessment of fish consumption for humans.

## 2. Results

### 2.1. Rationale for Method Selection

ELISAs are among the most intensively used methods for screening contaminants in different matrices. However, there are several difficulties for the analysis of MCs in fish by ELISA. The first difficulty arises because MCs are usually extracted with 60% or higher concentrations of organic solvents [34,36]. As the antigen–antibody binding is distorted by these concentrations of organic solvents, it is necessary to remove them before immunodetection. Furthermore, various components of the matrix could alter the analyte–antibody recognition, and thus a highly selective antibody is required to avoid immunoassay interference. Indeed, attempts to analyze unexposed fish extracts spiked with MC-LR using our previously developed polyclonal antibody have led to the relevant overestimation of the MCs (Table 1). This occurred despite the assay having been validated for the analysis of ambient waters in the range 0.3–2.5 µg/L [47].

The nanobody Nb A2.3, isolated from a llama antibody phage display library using an off-rate selection strategy, has a high affinity and a broad cross-reactivity for the most common MCs, as reported by Pírez et al., 2017 [45]. With this Nb, it was possible to set up a sensitive and selective ELISA for the analysis of water samples with a detection limit (DL) of 0.05 µg/L for MC-LR [45]. In fish (Table 1), this Nb-ELISA showed excellent MC-LR recovery (77–119%) and precision (<15%) throughout the range studied (0.1–5 µg MC-LR/L extract; 0.59–29 ng MCs/g fish wet weight).

As ELISA does not identify the individual variants, it is useful to have another simple method to allow for the determination of MC congeners such as MALDI-TOF [48,49,50]. Moreover, using an internal standard, MALDI-TOF MS operated in reflector mode is suitable for the rapid quantification of individual MC congeners directly in untreated ambient water samples with a DL of 2.8 µg/L and a quantification limit (QL) of 4.6 µg/L MC-LR [51]. These limits of detection and quantification would correspond in fish extracts to 16 and 27 ng MC-LR per gram of wet fish, respectively. An intake of 100 g of fish containing 27 ng/g MC-LR represents 113% of the TDI (0.04 μg MC-LR/kg × 60 kg = 2.4 µg MC-LR per person). Thus, to improve the quantification level, fish extracts should be concentrated before MALDI-TOF analysis. In the Nb-QMALDI MS, the samples were concentrated 50-fold by binding to the Nb immobilized to the magnetic beads. Subsequently, the congeners were identified by the *m*/*z* signals corresponding to the [M + H]^+^ ions present in the MALDI spectra.

Regarding the QL required for risk assessment, this depends on the fish consumption rate of the population, which is highly variable in different parts of the world [52]. A consumption of 100 g of fish and shellfish per day is equivalent to 36.5 kg per person per year. This consumption rate and occasionally higher values are found in many densely populated regions (Southeast Asia, China as well as northern and southern European countries), so we used this as a basis to evaluate the quantification levels required. Since the WHO has defined an allocation factor of 0.2 for MC exposure routes other than drinking water [27], we considered that the screening methods should be able to quantify at least 20% of the TDI for an intake of 100 g/fish per day, namely 5 ng MCs per g of fish. Since the Nb-ELISA and the Nb-QMALDI MS can potentially achieve this quantification level, we decided to evaluate both methods in fish extracts.

### 2.2. Validation of the Methods for the Analysis of MCs in Fish Extracts

#### 2.2.1. Nanobody Based ELISA

**Figure 1:**
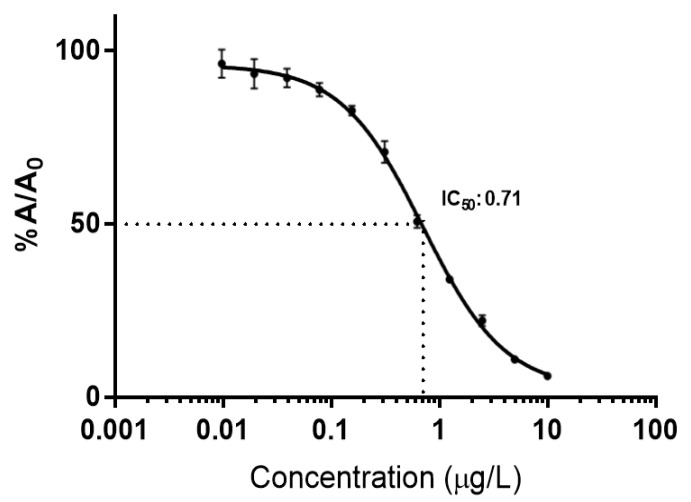
Calibration curve of the Nb-ELISA (*n* = 4, r^2^ = 0.999). % A/A_0_ = % of absorbance of the zero concentration. The concentration range was 0.01–10 µg/L MC-LR and the calculated IC_50_ (half maximal inhibitory concentration) = 0.71 µg/L (95% confidence interval = 0.63–0.81 µg/L).

The typical calibration curve of the Nb-ELISA was fitted to a sigmoidal 4 parameter logistic curve (4PL) in the range 0.01–10 μg/L MC-LR (Figure 1). The 50% inhibitory concentration value (IC50) was 0.71 μg/L and the r^2^ = 0.999.

The results of the initial demonstration of capability (precision, accuracy, confirmation of the method reporting limit (MRL)) and other validation parameters of the Nb-ELISA in fish extracts are reported in Table 2. The method reporting limit (MRL = 0.3 µg/L) was confirmed by the analysis of seven fortified unexposed fish extracts at this concentration, as both upper and lower prediction interval of results (PIR) values were within the requirements of the U.S. EPA Method 546 for the determination of the total MCs and nodularins in water by Adda-ELISA [42]. As required, the laboratory reagent blanks analyzed throughout the plate represented less than one-half of the MRL. In our extract (0.17 g fish/mL), this MRL value corresponded to 1.8 ng MC-LR per gram of fish wet weight (ng/g fish ww). An ingestion of 100 g of fish per day containing this concentration represents 7.5% of the WHO TDI during a lifetime (2.4 µg per person and day), so it is very adequate for the risk analysis of fish consumption. As shown in Table 1, the matrix effects or spurious (unspecific) cross reactivities were not observed, thereby, the assay range of the Nb-ELISA that showed recovery rates within 70–130% and RSD values lower than 15% in the fish extracts resulted in being 0.3–5 µg/L MC-LR (1.8 to 29 ng MC-LR/g fish ww). For an ingestion of 100 g of fish per day, this assay range corresponded to 7.5–121% of the MC-LR TDI. Samples with higher concentrations should be diluted to fit the assay range.

#### 2.2.2. Immuno-Concentration in Magnetic Beads with Immobilized Nb Q-MALDI MS

The results of precision, accuracy, confirmation of MRL, and other validation parameters for the Nb-QMALDI MS were calculated from seven fortified lab matrix blanks (Table 3). The confirmed MRL of the four most common MC-variants in the study area (LR, RR, YR, and WR) resulted in 0.05 μg/L for each MC variant (0.29 ng/g fish). This MRL is certainly very low, which is important in a method that quantifies individual congeners to facilitate the identification of MCs in fish samples with a sum of variants of 5 ng/g fish. It is noteworthy that the calibration curves were very similar for the four MC variants tested, and consequently, they all have the same MRL. The total assay range (0.05–5 μg/L) corresponded to 0.29–29 ng/g fish for each variant. The accuracy was in the range of 87–121% and the precision was in the range: 3.1–5.1% for MC-LR, MC-RR, MC-YR, and 4.0–7.3% for MC-WR. 

### 2.3. Analysis of Water and Fish

#### 2.3.1. Water Samples

**Figure 2:**
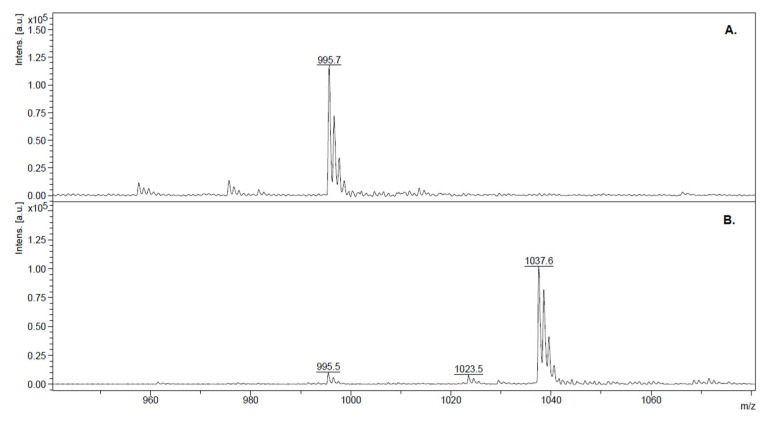
MALDI-TOF spectra of water samples from Palmar Dam in both campaigns. Peaks with *m*/*z* corresponding MCs are labeled. (**A**) February 2017, *m*/*z* = 995.6 (MC-LR); (**B**) March 2021 *m*/*z* = 995.6, 1023.5, and 1037.6 (which correspond to the single protonated ion of MC-LR; [D-Leu^1^, Dha^7^] MC-LR; and [D-Leu^1^] MC-LR).

Palmar is a eutrophic hydroelectric dam reservoir (320 km^2^) in the Negro River, Uruguay, South America, with frequent MC-producing cyanobacterial blooms between January and May, corresponding to the Southern Hemisphere’s summer and fall [53,54]. The fish analyzed were captured from Palmar Dam in two campaigns (February 2017 and March 2021). The results of the water analysis of 2017 and 2021 by ELISA evidenced the presence of blooms with high concentrations of MCs in both campaigns (850 and 4600 μg/L, respectively). In 2017, the MALDI-TOF MS spectra of Palmar showed a main peak that corresponded to the *m*/*z* of the singly protonated ion from MC-LR, and two other minor peaks that did not match with any of the close to 300 MC variants *m*/*z* listed by Bouaïcha and coworkers [12] (Figure 2A). In 2021, the major peak detected had a *m*/*z* of 1037.6 (Figure 2B), which, among the other possible variants, could correspond to the [M + H]^+^ ion from [D-Leu^1^] MC LR, previously identified in nearby watersheds, both in Argentina and Brazil [55,56].

#### 2.3.2. Fish Samples

**Figure 3:**
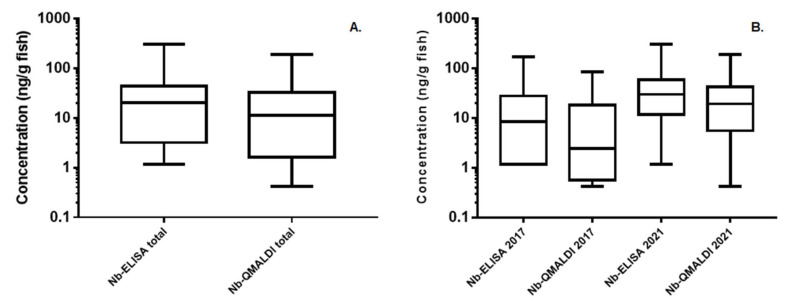
Boxplots for the MC analysis in fish, classified by method and campaign (*n* = 55). (**A**) All data classified by method (Nb-ELISA and Nb-QMALDI); (**B**) Nb-ELISA and sum of congeners by Nb-QMALDI MS in ng/g fish wet weight, classified by campaign.

The MC results in fish by ELISA and the sum of congeners by Nb-QMALDI MS in both individual sampling campaigns (2017, 2021) as well as all the data together, are summarized in Figure 3 and the supporting data are reported in Supplementary Materials Table S1. In all cases (2017, 2021, and total), the medians of ELISA (8.4, 30.2, 20.2 ng/g fish ww) were higher than those of Nb-QMALDI MS (2.4, 19.2, and 11.3 ng/g fish ww). To perform a statistical comparison of the MC results in fish analyzed by ELISA and Nb-QMALDI MS as well as for the same method between campaigns, it was necessary to inquire into the normality and homoscedasticity of all data groups. As the normality test was negative for all groups (KS *p* < 0.0001), the data were transformed by the natural logarithm. Then, the normality (KS *p* > 0.1000) and homoscedasticity (F test *p* = 0.3802 and Bartlett test *p* = 0.6185) of the data were confirmed. One-way analysis of variance (ANOVA, Tukey’s multiple comparisons test) was used to test the difference between the means of the results. These tests did not show significant differences between methods for all data (*p* = 0.3511) and for each campaign (2017: *p* = 0.5765; 2021: *p* = 0.8395). The consistent trend suggests that the variability of the results is not method dependent. Regarding the comparison between campaigns by the same method, for ELISA, we could not conclude that there were significant differences, although the *p* value was close to 0.05 (*p* = 0.0677). On the other hand, for the Nb-QMALDI MS, the differences between campaigns were significant (*p* = 0.0152). Thus, the year-to-year variability could be due to the sampling timing (e.g., the 2021 bloom was more intense than the 2017 bloom), fish species, and cyanobacterial blooms (e.g., different MC variants present).

**Figure 4:**
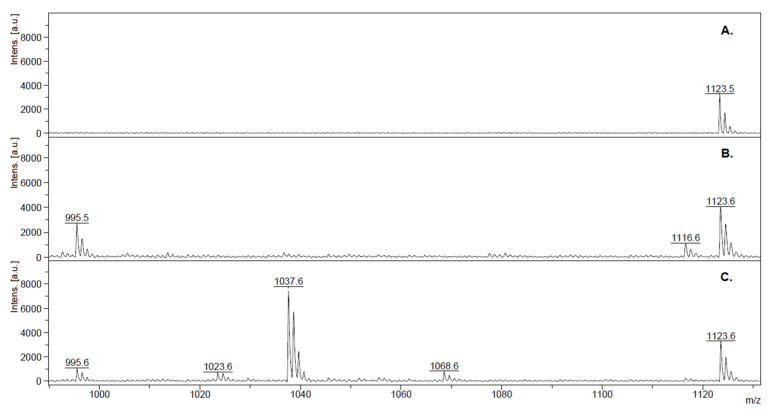
Nb-QMALDI MS of the fish samples. Typical spectra of fish samples from Palmar in both campaigns analyzed. The *m*/*z* = 1123.6 corresponds to the internal standard used for quantification. (**A**) Unexposed fish (LMB); (**B**) February 2017 sample with MCs: *m*/*z* = 995.5 and 1116.6 (which correspond to the single protonated ion of MC-LR and MC-LR-Cys); (**C**) March 2021 sample with MCs: *m*/*z* = 995.6, 1023.6, 1037.6, and 1068.5 (which correspond to MC-LR, [D-Leu^1^, Dha^7^] MC-LR, [D-Leu^1^] MC-LR, and MC-WR, respectively).

In agreement with the water results, the Nb-QMALDI MS in fish showed that the *m*/*z* of the single protonated ion of the MC-LR was the major and almost the only variant present in 2017, while the MC corresponding to 1037.6 *m*/*z* was predominant in 2021 (Figure 4). Other minor peaks present in some samples from 2021 (1068.6, 1038.6, and 1045.5 *m*/*z*) corresponded to the [M + H]^+^ ion of MC-WR, MC-RR, and MC-YR, respectively. In both campaigns, peaks corresponding to singly protonated ions of the cysteine conjugates of the major variants were observed (1116.5 and 1158.5 *m*/*z*). These conjugates were detected in 44% of the total number of samples (*n* = 55) and on average, they represented 14% of the sum of all congeners. Supplementary Materials Figure S1 shows that in the *m*/*z* range 900–1370, no additional peaks were observed.

### 2.4. Correlation ELISA—Sum of MC Congeners by Nb-MALDI MS in Fish Extracts

**Figure 5:**
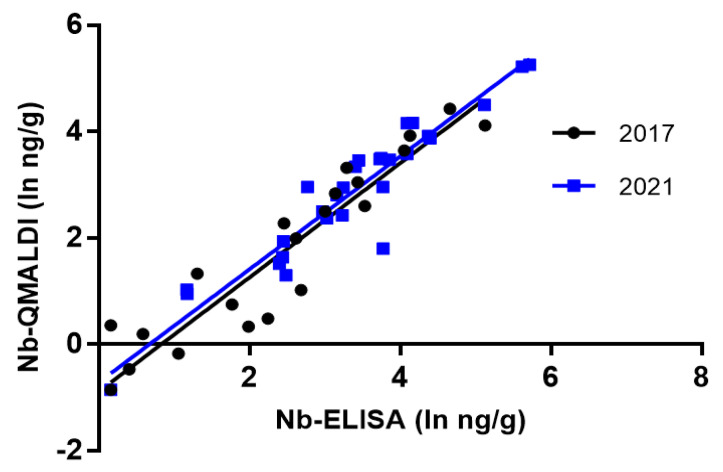
Correlation between the methods of MC analysis in fish: ELISA and the sum of congeners by Nb-QMALDI MS for each campaign (2017 *n* = 26 and 2021 *n* = 29). Ln (concentration ng/g fish) is the variables x and y for normalization.

To evaluate the possible application of ELISA as a screening method in fish, it is critically important to verify that it effectively provides results on the MC variants and not on other possible cross-reactive compounds. Therefore, we studied the correlation between the MC results by ELISA and the sum of congeners quantified by the Nb-QMALDI MS method. The linear correlation between methods was studied for all the data together and for each campaign. The correlation for all data (2017 and 2021 campaigns) was very strong: r Pearson = 0.9637 (*p* < 0.0001) and r Spearman: 0.9645 (*p* < 0.0001). This strong correlation suggests that ELISA provides information that accounts for the MCs present in the sample, and thus that it is potentially useful as a screening test. The correlation for each sampling campaign is shown in Figure 5 and Table 4. The coefficient of determination was very strong in both cases (r^2^ = 0.92), so there was a high goodness of fit to the linear model in both campaigns. Furthermore, there were no significant differences (F test) between the slopes and the intercepts of both campaigns (*p* values 0.9008 and 0.3117, respectively). This result is remarkable considering that the MC variants found in the fish of both seasons were different: in 2017, MC-LR was the main variant and in 2021, the *m*/*z* corresponding to the single protonated ion of [D-Leu^1^] MC-LR was dominant and occurred together with other minor MC-variants (Figure 4). These results strongly suggest that ELISA could be used to predict the sum of MC congeners by Nb-QMALDI MS, and therefore we continued testing this hypothesis by ROC analysis.

### 2.5. Receiver Operating Characteristic (ROC) Curves

**Figure 6:**
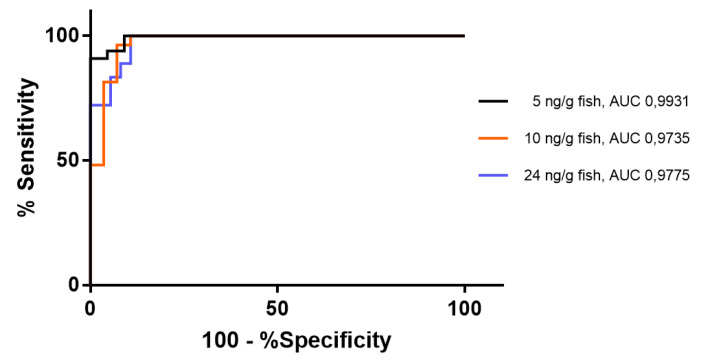
ROC curves for the Nb-ELISA as a test for the prediction of three threshold-values of the sum of all variants by Nb-QMALDI MS. AUC = Area under the curve.

To evaluate the performance of ELISA as a screening test for risk assessment, the ELISA data were evaluated as a predictor of the sum of MC congeners by Nb-QMALDI MS using the ROC curves. Since there are no generally accepted guidelines for the maximum concentration of MCs in seafood to define the positive and negative samples in the ROC analysis, three threshold levels for the sum of all MC congeners were tested. The first threshold level selected was 5 ng/g fish ww (close to 20% of the TDI for a daily intake of 100 g of fish/person), which corresponded to the WHO allocation factor for other routes of MC intake, in addition to drinking water [27]. The other two thresholds selected were 10 and 24 ng/g fish ww, which corresponded to 40% (twice the above-mentioned allocation factor) and 100% of TDI, respectively. The latter two threshold levels would be particularly important for vulnerable populations and those with a high rate of fish consumption. The ROC curves are plotted in Figure 6 and the values that maximized the specificity while keeping 100% of the sensitivity are detailed in Table 5. A high sensitivity is important to minimize false negatives (samples with a sum of MCs higher than the threshold selected that are not detected by ELISA). The area under the curve (AUC) represents the overall performance of the binary classifier. The highest AUC was observed for the threshold of 5 ng/g fish (0.9931), followed by the 24 ng/g fish (0.9775) and 10 ng/g fish (0.9735). In all cases, the AUC was much higher than 0.9, which is considered as outstanding [57]. To achieve a 100% sensitivity (negative predictive value; NPV = 1) and maximum specificity, the resulting ELISA cut-off values for detecting the sum of MC congeners of 5, 10, and 24 ng/g fish were respectively: 11.5 ng/g (91% specificity); 15.3 ng/g (89% specificity); and 26.3 ng/g (89% specificity). The very high specificity attained with 100% sensitivity and the corresponding positive predictive value (PPV) (0.94, 0.90, and 0.82, respectively) show that Nb-ELISA can be a highly promising screening method to improve the food safety of fish consumption. The lower prevalence of samples with higher values of MCs is most probably the reason why the PPV decreases with the increase in the threshold tested [58,59].

Following the recommendations of the WHO [27], the sum of all congeners found including the cysteine metabolites were used as a conservative approach for this analysis. However, since it has been argued that the toxicity of the cysteine conjugates, which are detoxification metabolites, is 3–9 lower than the parent toxins [60], we also analyzed the accuracy of the prediction for the sum of all free toxins excluding these conjugates. In this case, the ELISA cut-off values for the prediction of the three selected thresholds were the same for the 10 and 24 ng/g fish and very close to the previous analysis for 5 ng/g fish (cut-off = 11.2 ng/g fish). Moreover, the maximum specificity values for a 100% sensitivity were the same as for the sum of all congeners including the cysteine metabolites, and the AUC values were 0.9902, 0.9643, and 0.9775 for the prediction of 5, 10 and 24 ng/g fish, respectively. Thus, the presence of metabolites does not significantly affect the prediction of the free toxins.

## 3. Discussion

It is well-known that MCs can be transferred through food webs and accumulate in fish, where they undergo multiple bio-transformations (detoxification, covalent binding to proteins, etc.). MCs are present in animal tissues both as free toxins as their soluble detoxification metabolites (such as gluthatione- and cysteine-conjugates) as well as covalently bound to proteins [60]. Free toxins are expected to be more readily bioavailable, so most protocols for the determination of MCs in biological tissues involve extraction with high concentrations of organic solvents (as in our case, acetonitrile: water 75:25). Under these conditions, the MCs covalently bound to proteins precipitate, and therefore free MCs as well as several low molecular weight metabolites are extracted. The free MCs and the detoxification conjugates are hepatotoxic and have been shown to travel up the aquatic food web [61]. These bio-transformations and the lack of analytical standards for most of the close to 300 known MC congeners complicate the analysis and limit the availability of reliable data on their occurrence in fish and shellfish as well as the evaluation of risks for human consumption. MC monitoring in water has been simplified by ELISA, which has allowed laboratories in low-resource settings worldwide, to obtain data that lead to the prevention of exposure through drinking and recreational water. This is not the case for the analysis of MCs in seafood. The current analytical methods are too complex and diverse to draw global conclusions from the existing data, so there is a need to develop both standard reference methods as well as simple screening tools. For screening purposes, immunodetection is affordable and simple, but it could be troublesome due to matrix effects and cross-reactivity with detoxification metabolites. To overcome these limitations, we studied the performance of NbA2.3 for the analysis of MCs in wild fish using two previously described methods.

Nanobodies are small sized (15 kDa) advantageous analytical immuno-reagents that have shown excellent analytical performance in complex matrices [62,63]. The latter is critical for the analysis of MCs in food products and its relevance was made evident in the example of poor performance of the polyclonal antibody shown in Table 1. As argued above, the good results obtained with NbA2.3 could be related to a smaller antigen binding site. In comparison to conventional antibodies, this limits the reactive surface exposed to the solvent after the binding of their cognate small analytes, decreasing the possibility of spurious reactivity against occasional components of complex matrices.

In this paper, we presented an example of the use of an anti-MCs Nb in ELISA and in quantitative MALDI-TOF (Nb-Q MALDI MS) as a simple strategy to facilitate the generation of data and to accelerate the understanding of cyanotoxin transfer through the food web. Both methods are fast and have a low operating cost. As a thorough evaluation of the methods is essential, we validated both methods and used them to analyze a set of 55 fish samples from a eutrophic dam taken in two different years (2017 and 2021). The results by both methods were strongly correlated (r^2^ of the ln transformed data = 0.92), and the correlation parameters (slope and y-intercept) were not distinguishable (F test *p* = 0.9008 and 0.3117, respectively), regardless of the sampling campaign and of the variants present. Using ROC analysis, we showed that ELISA could be used to predict, with 100% sensitivity and very high specificity (>89%), the sum of all MC congeners (both including and excluding the detoxification metabolites) analyzed by Nb-Q MALDI MS, for samples containing more than 5, 10, and 24 ng/g fish ww (20%, 40%, and 100% of the TDI for an intake of 100 g fish). As free and thiol-conjugated MCs can travel up the aquatic food web, both methods could be used to detect risks in a conservative way for all the variants including the metabolites [64,65].

Finally, the median of the sum of all variants for the 55 samples analyzed by Nb-Q MALDI in the fish of Palmar Dam (11.30 ng/g ww) was equivalent to 47% of the TDI for a daily intake of 100 g of fish. This indicates a potential concern for public health, which means that more research is needed to find answers to basic questions such as how long this situation persists; what are the species and sizes that accumulate higher concentrations; and what is the actual risk of exposure considering the consumption rate of these species/sizes? To answer these questions, it is essential to implement an intensive monitoring program of water and fish to study the spatial and temporal variability of the MC occurrence in fish in this dam and other similar ecosystems using the screening methods developed (ELISA, Nb-QMALDI MS). Additionally, a subset of the samples should be analyzed by reference untargeted and targeted LC-MS methods to unequivocally identify and quantify the occurrence of the different MC variants. These recommendations are particularly relevant considering the possible role of MC-LR as a carcinogen [22] as well as other serious possible chronic effects in the reproductive system, kidney, and intestine [23,24,25].

## 4. Conclusions

Both Nb-based methods, ELISA and Nb-QMALDI MS, are sensitive and showed excellent performance in fish extracts, which makes them auspicious as simple tools to analyze MCs in muscle tissue across the food chain. They can be used independently or in tandem to investigate and assess the MC variants present in the selected samples. We have shown that ELISA can be useful to predict the presence of different thresholds (5, 10, and 24 ng MCs per g of fish ww) with no false negatives and specificity >89%.The fish with 5 ng/g MCs should represent no risk for consumers with no other sources of exposure, even for populations with a high fish consumption rate of 100 g per capita per day during their lifetime (36.5 kg fish in a year). Other simpler formats for using the Nb such as immuno-chromatography strips may be worth testing, which could be extremely helpful in the routine monitoring of market seafood. Finally, since one of the main purposes of this work was to contribute with tools for the study of fish consumption safety, it is worth noting that the NbA2.3 sequence is public [45], and therefore the critical element of the methods reported in this work can be reproduced in any laboratory worldwide.

## 5. Materials and Methods

### 5.1. Chemicals and Reagents

Organic solvents (Hexane, Acetonitrile) were HPLC grade from J.T. Baker (Phillipsburg, NJ, USA). Microcystins (LR; RR; YR; WR) were from Enzo Life Sciences (Farmingdale, New York, NY, USA) and the MC-LR Certified Reference Standard (CRM-MCLR) from National Research Council, Ottawa, ON, Canada. Alfa-cyano-4-hydroxycinnamic acid (CHCA) and peptide calibration standards (low molecular weight) were obtained from Bruker Daltonics (Frederikssund, Denmark). MagnaBind streptavidin beads were from ThermoFisher, MA, USA. Bovine serum albumin (BSA) was obtained from Golden West Biologicals (Temecula, CA, USA) streptavidin-peroxidase (HRP) conjugate, SPO (Thermo Fisher Scientific Inc., Waltham, MA, USA); β-mercaptoethanol (99.9%). All other reagents were obtained from Sigma-Aldrich (St. Louis, MO, USA).

### 5.2. Fish and Water Sampling

Two sampling campaigns in February 2017 and March 2021 were conducted at Palmar Dam at the Negro River (33°4′13.22″ S, 57°27′40.04″ W), on one spot with almost constant cyanobacterial blooms during summer and autumn (January–April). Using fishing nets, 20 and 18 different species were sampled in each campaign, respectively. The specimens were counted, cleared out of their digestive organs, weighed, and measured for length. Water samples were taken at a depth of 20–30 cm at the same time and sample spot of the fish collection. The fish and water samples were individually frozen and kept at −20 °C until extraction.

### 5.3. Microcystin Extraction

Microcystins (MCs) in the water samples were released from the cyanobacterial cells by three freeze–thaw cycles and the samples were filtered through a 1 µm pore glass-fiber syringe filter. Individual fish samples were homogenized on a crusher and HG-15A homogenizer (Daihan, Korea). A fraction of 1.7 g (wet tissue) was extracted with 10 mL of acetonitrile: water 75:25 *v*/*v*. Fish extracts were defatted by liquid–liquid partition using hexane saturated with acetonitrile [34], and then the solvent was evaporated in a Speedvac concentrator device model SPD1010-230 (Thermo Fisher Scientific, MA, USA) at 45 °C. The extracts were then brought to a final volume of 10 mL with ultrapure type I lab water (Milli-Q^®^ water purification system model SIMSV00WW, Merck Millipore, MA, USA). The same extraction procedure was performed with reagent water, as the laboratory reagent blank (LRB); with unexposed fish (*Astraloheros facetus*) as the laboratory matrix blank (LMB); and also with the fortified unexposed fish as the laboratory fortified sample matrix and duplicate (LFSM and LFSMD) for their use in the initial demonstration of capability, validation, and quality control requirements of the U.S. EPA microcystin determination methods 544 (for LC-MS/MS) and 546 (for ELISA).

### 5.4. Analysis of Microcystins

#### 5.4.1. Nanobody Based ELISA

Fish extracts were analyzed by ELISA using a single domain antibody fragment (Nanobody) from a llama heavy chain monoclonal antibody, as described in Pírez-Schirmer et al. (A2.3 clone) [45]. The recombinant nanobody was produced in E. coli and in vivo biotinylated to facilitate detection using the streptavidin-peroxidase (SPO) conjugate.

The ELISA plates were coated overnight with a MC-BSA conjugate (60 μg/L) at 4 °C and blocked using 0.5% gelatin *m*/*v* (Sigma-Aldrich, St. Louis, MO, USA) in PBS. An aliquot of 150 µL of the sample or MC-LR standard solution (seven standards in the range 0.16–5 µg/L in phosphate buffered saline solution, PBS) was preincubated with 33 µL of 1 M Tris, 0.3 M NaCl, 0.2 M EDTA, 1% BSA, pH 7.4 buffer. Then, 50 µL/well was transferred to the ELISA plate (triplicates) and combined with 50 µL of the biotinylated nanobody (7 ng/mL), incubated for 1 h, and washed. Subsequently, the wells were incubated with 100 µL of HRP-peroxidase, SPO solution, and after extensive washing, the peroxidase activity was measured by the addition of 100 µL of TMB peroxidase substrate (0.4 mL of 6 mg of 3,3′,5,5′-tetramethylbenzidine in 1 mL of DMSO with 0.02 mL of 6% H_2_O_2_ in water, in a total of 25 mL of 0.1 M acetate buffer, pH 5.5). After 15 min, the enzyme reaction was stopped by the addition of 50 µL of 2 N H_2_SO_4_, and the absorbance was read at 450 nm using a Fluostar Optima Reader (BMG, Ortenberg, Germany).

The calibration curves were plotted using the ratio absorbance of each standard and the zero-absorbance expressed in % (%A/A_0_) versus the MC–LR concentration and fitted with a sigmoidal 4PL model from the GraphPad Prism 7 software (curves with r^2^ higher than 0.98 were accepted).

Samples were analyzed in triplicate, in at least two dilutions. Each batch (18 samples) contained the calibration curve, one quality control standard (QC, 1 µg/L), two negative controls (LRBs), one low-calibration verification control (LRB at fortified at 0.2 µg/L), two calibration verifications (LRB fortified at 0.4 and 5 µg/L), and one certified reference standard at 1 µg/L were performed and evaluated according to the U.S. EPA method 546 requirements. Additionally, the extraction controls of the unexposed fish spiked at 10 ng/g fish ww (LFSM and LFSMD) were analyzed and the resulting recoveries were in the range of 52–65% with a relative percent difference between replicates (RPD) lower than 40%, as specified in the method.

#### 5.4.2. Immuno-Concentration in Magnetic Beads with Immobilized Nb (Nb-QMALDI MS)

Fish extracts were also analyzed by MALDI-TOF/MS based on the method previously reported by Pírez Schirmer et al., 2019 [46]. Briefly, samples and MC standards were captured and concentrated in magnetic beads, with previously immobilized anti-MC Nb A.2.3, and the internal standard (IS: MC-YR derivatized with β-mercapto-ethanol) to facilitate quantification. One mL of each of the standards and samples was incubated for 15 min with 5 µL of a streptavidin magnetic bead suspension containing 50 µg of beads (binding capacity 4644 pmoles of fluorescein labeled biotin/mg of beads) in 0.2% bovine serum albumin-phosphate buffered saline, PBS-BSA. Then, the beads were washed twice with PBS-Tween 0.1% and 2 Water-Tween 0.002% (*v*/*v*). Beads were collected and resuspended in 10 µL of a water 0.1% trifluoroacetic acid and acetonitrile (50:50, *v*/*v*) solution and after 10 µL of MALDI matrix, α-cyano-4-hidroxicinamic acid (CHCA) was added. In these conditions, the MCs captured in the beads allowed for both extract purification and 50-fold concentration. Magnetic beads were directly dispensed in the MALDI plate in triplicate and dried off at ambient temperature. Analysis was carried out using Microflex LRF MALDI-TOF/MS (Bruker Daltonics, Billerica, MA, USA) equipment, operated in positive mode, with reflector and retarded extraction using a nitrogen laser of 337 nm. The instrument was optimized between a 500 and 2000 *m*/*z* signal and a laser intensity of 50%. The laser was shot 250 times at eight different places per spot (2000 shots per spot).

The tentative identification of congeners and data collection were performed based on the *m*/*z* of the single protonated ion [M + H]^+^ of the MCs listed in Bouaïcha et al. [12] and in Section VIII (Appendix 3) of the Handbook of Cyanobacterial Monitoring and Cyanotoxin Analysis [13]. For the *m*/*z* that corresponds to several possible congeners, we tentatively identified them as the most frequent variant. An unequivocal identification or structure elucidation was beyond the scope of a simple and fast method such as that hereby proposed. The results of the Nb-QMALDI MS method are expressed as the sum of the tentatively identified congeners.

Calibration curves were performed with five concentrations of a multi-standard mix of MC-LR, RR, YR, and WR in two ranges: 0.05–0.7 µg/L and 0.7–5.0 µg/L. Samples and standards were analyzed in triplicate spots, and calibration curves were performed by plotting the ratio of the ion intensity for each MC *m*/*z* detected and the intensity of the IS.

The following quality controls were performed per batch of 20 samples: one LRB, one LFB fortified at 0.1 µg/L, one CRM (0.5 µg/L) as well as two extraction controls of unexposed fish fortified at 10 ng MC LR/g fish ww (LFSM and LFSMD). The results of the mean recovery of the LFSM duplicates were in the range 64–82% with a relative percent difference between replicates (RPD) lower than 40%, as specified in the USA EPA method 544.

### 5.5. Methods Validation

Laboratory reagent blanks and laboratory matrix blank (LRB and LMB) were spiked for recovery analysis following the U.S. EPA guidelines for MC analysis [42,66]. For Nb-ELISA, seven replicates of LMB were spiked in three concentrations (0.1, 0.3, 1.0 µg/L MC/LR) and analyzed. The detection limit (DL) was calculated as the product of the standard deviation of 0.1 µg/L with 16 spiked replicates in LMB and the t statistic for *n*−1 degrees of freedom with 0.05% of significance.

The MC-LR spiked recovery experiment with a polyclonal antibody was performed with the polyclonal antiserum and ELISA method reported in [47].

For Nb-QMALDI MS, seven replicates of LRM were spiked with the MC multi-standard mix of MC-LR, RR, and YR at three concentrations of each variant (0.05, 1.0, and 3.0 µg/L) and analyzed. DL was calculated as the product of standard deviation of 0.025 µg/L spiking 18 replicates in LMB with the t statistic for *n*−1 degrees of freedom with 0.05% of significance.

### 5.6. Data and Statistical Analysis

Calibration curve plotting and data interpolation were carried out with GraphPad Prism 7 software. For nanobody ELISA, fitting was achieved with a 4PL sigmoidal model, the most common regression model used to analyze bioassays such as ELISA. The equation is as follows:

(1) $$y=Bottom+\frac{Top-Bottom}{1+{\left(\mathrm{IC}50/x\right)}^{\;\left(Hill\;slope\right)}}$$

where *y* = % absorbance (% of the zero concentration); *x* = concentration of MC-LR; *Top* = % absorbance at the top plateau; *Bottom* = % absorbance at the bottom plateau; *Hill slope* = slope at the inflexion point; and *IC*_50_ = concentration at the inflexion point.

For the Nb-QMALDI, calibration curve modeling was fitted by a linear regression in two ranges (0.05–0.7 and 0.7–5.0 µg/L).

The statistical tests (normality, homoscedasticity, ANOVA) and ROC curves were performed with GraphPad Prism 7 software. For normality analysis, we employed the Kolmogorov–Smirnov test, and for homoscedasticity, the Bartlett test. For the comparison of the data subgroups, an ANOVA one way test (Tukey´s multiple comparisons test) was applied to compare the means.

## Figures and Tables

**Table 1:** Recovery of MC-LR in the unexposed fish extract by ELISA.

		Nanobody ELISA		Polyclonal ELISA	
MC-LR		Recovery	RSD	Recovery	RSD
μg/L	ng/g Fish	%	%	%	%
5.0	29	77	14	118	34
2.5	15	80	14	116	27
1.5	8.8	84	13	130	29
0.60	3.5	84	7	162	32
0.30	1.8	84	12	215	44
0.15	0.88	89	10	333	43
0.10	0.59	119	8	483	56

Results are the average of four determinations. RSD: Relative standard deviation.

**Table 2:** Nb-ELISA: Validation parameters for the analysis of MCs in the fish extracts.

Parameter	Methodology/Calculation	Result	Acceptance Criteria *
Precision and Accuracy	7 replicate lab fortified blanks (LFBs) at 1 µg/L MC-LR	Rec. = 93.9%; RSD = 5.2%	70% < Recovery < 130% RSD < 15%.
DL µg/L (ng/g fish ww)	SD × t (*n*−1 = 15, 1−α = 0.99) *n* = 16	0.079 µg/L (0.46 ng/g)	
Quality Control-Std at 1 µg/L (SD)	Certified Standard from NRC Canada, CRM-MCLR	0.91 µg/L (SD = 0.07 µg/L)	70% < Recovery < 130%
Method Reporting Limit (MRL) Confirmation (ng/g fish ww)	7 replicates lab matrix blank (LMB) fortified at 0.3 μg/L MC-LR	0.3 µg/L (1.8 ng/g fish ww)	<0.85 µg/L (5 ng/g) **
Upper PIR Limit	$=\frac{Mean+HR_{PIR}}{Fortified\;concentration}\times 100$ $HR_{PIR}=3963\;\times \;s$	143.9%	≤150%
Lower PIR Limit	$=\frac{Mean-HR_{PIR}}{Fortified\;concentration}\times 100$ $HR_{PIR}=3963\;\times \;SD$	57.8%	≥50%
% Rec. 0.3 µg/L (RSD)	Average recovery of 7 replicates, LMB, fortified at 0.3 µg/L MC-LR	100.8% (RSD = 10.9%)	

DL = detection limit; SD = standard deviation; t = Student test (99% confidence *n*−1 degrees of freedom). Rec. = recovery; RSD = relative standard deviation; PIR = prediction interval of results. * U.S. EPA Acceptance criteria ** Acceptance criteria based on 20% TDI for 100 g fish, as explained in the text.

**Table 3:** Nb Q-MALDI MS: Validation parameters for the analysis of the MCs in fish extracts of the MC congeners: MC-LR, MC-YR, MC-RR, and MC-WR.

Parameter	MC-LR (μg/L)	MC-RR (μg/L)	MC-YR (μg/L)	MC-WR (μg/L)	Acceptance Criteria *
DL (*n* = 18)	0.02				
MRL	0.05	0.05	0.05	0.05	<5 ng/g **
Upper PIR	131.4	116.3	143.1	144.1	≤150%
Lower PIR	95.2	90.4	95.2	98.9	≥50%
% Rec. 0.05 µg/L (RSD)	113	103	119	121	50–150%
	(4.0)	(3.2)	(5.1)	(4.7)	<50%
Calibration Curve Low range 0.05–0.7μg/L	y = 5.331x − 0.112	y = 5.233x − 0.120	y = 4.841x − 0.098	y = 3.451x − 0.042	
r^2^	0.9955	0.9957	0.9955	0.9975	
High range 0.7–5.0 µg/L	y = 5.539x − 0.287	y = 5.918x − 0.514	y = 5.030x − 0.102	y = 4.111x − 0.076	
r^2^	0.9988	0.9981	0.9991	0.9967	
% Rec. 1 µg/L (RSD)	106	106	101	93	70–130%
	(3.5)	(3.8)	(4.1)	(7.3)	<30%
% Rec. 3 µg/L (RSD)	104	107	101	87	70–130%
	(4.1)	(4.5)	(4.1)	(4.0)	<30%

DL = detection limit; Rec. = recovery; RSD = relative standard deviation; PIR = prediction interval of results. The calculations were made as reported in Table 2. * U.S. EPA Acceptance criteria ** Acceptance criteria based on 20% TDI for the ∑ MCs per 100 g fish, as explained in the text.

**Table 4:** Correlation parameters between the MCs in fish (ln ng/g fish) by ELISA and Nb-QMALDI MS (sum of variants) by sample campaign.

	2017	2021
Equation	y = 1.074x − 0.893	y = 1.063x − 0.718
r^2^	0.9162	0.9166
Number of values (*n*)	26	29
95% Confidence Intervals		
Slope	0.9373 to 1.211	0.9362 to 1.189
Y-intercept	−1.244 to −0.5418	−1.162 to −0.2727

**Table 5:** Results of the ROC analysis for the three threshold-values that maximize sensitivity and specificity for the use of ELISA to detect the sum of all MC congeners by Nb-QMALDI MS.

ELISA Cut-Off Value/Threshold Value Sum of MCs	Calculation	11.5 ng/g ELISA/ 5 ng/g *∑MCs*	15.3 ng/g ELISA/ 10 ng/g *∑MCs*	26.3 ng/g ELISA/ 24 ng/g *∑MCs*
% of Tolerable Daily Intake	% of 2.4 μg/capita	20%	40%	100%
Sensitivity	=TP/TP + FN	1.00	1.00	1.00
Specificity	=TN/TN + FP	0.91	0.89	0.89
Positively Predictive Value (PPV)	=TP/TP + FP	0.94	0.90	0.82
Negatively predictive Value (NPV)	=TN/TN + FN	1.00	1.00	1.00
Prevalence	=TP + FN/(TP + FP + TN + FN)	0.60	0.49	0.33

TP = True Positives; TN = True Negatives; FP = False Positives; FN = False Negatives.

## Data Availability

The data presented in this study are available in this article or Supplementary Materials.

## Supplementary Materials

The following supporting information can be downloaded at: https://www.mdpi.com/article/10.3390/toxins15020084/s1, Figure S1. Nb-QMALDI MS of the fish samples. Typical spectra of the analyzed fish samples from Palmar in both campaigns. Extended *m*/*z* range of the spectra shown in Figure 4. (A) Unexposed fish (LMB); (B) February 2017 sample with MCs: *m*/*z* = 995.5 and 1116.6 (which correspond to the single protonated ion of MC-LR and MC-LR-Cys); (C) March 2021 sample with MCs: *m*/*z* = 995.6, 1023.6, 1037.6, and 1068.5 (which correspond to MC-LR, [D-Leu^1^, Dha^7^] MC-LR, [D-Leu^1^] MC-LR, and MC-WR, respectively). Table S1. Data of the MC analysis in fish by Nb-ELISA and Nb-QMALDI MS (2017 and 2021).
