# Supplementary material for: Determination of Microcystins in Fish Tissue by ELISA and MALDI-TOF MS Using a Highly Specific Single Domain Antibody
Source: Toxins (Basel). 2023 Jan 17;15(2):84. doi: 10.3390/toxins15020084 (PMC9966346; doi:10.3390/toxins15020084)
Supplement: Supplementary file 1 [file toxins-15-00084-s001.zip › toxins-2112173-supplementary.pdf]

toxins

MDPI

Supplementary file

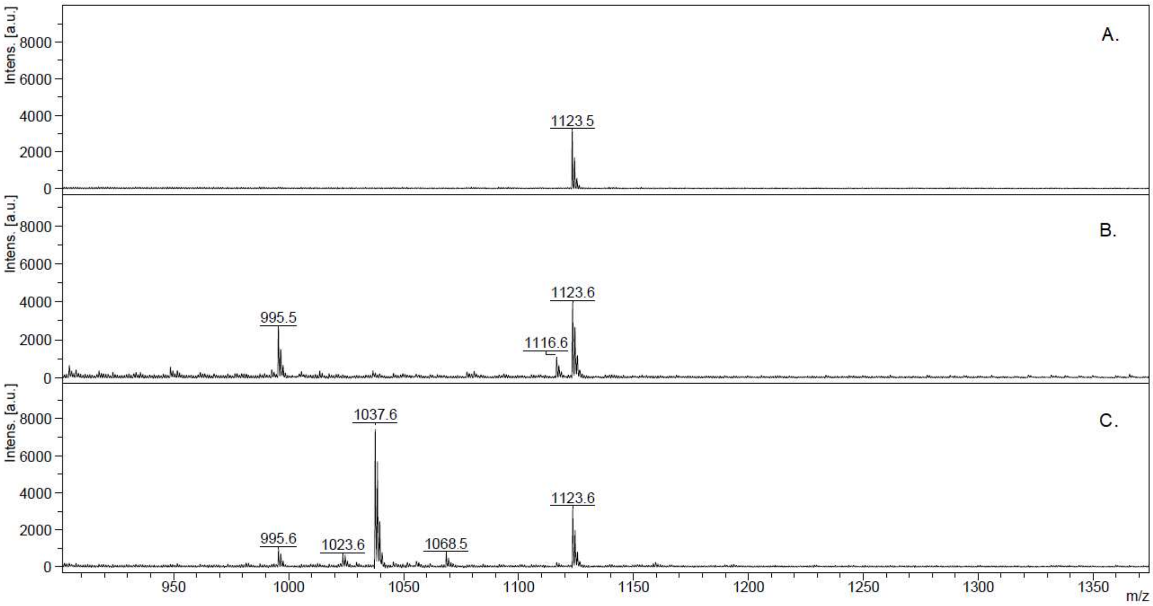

Figure S1. Nb-QMALDI MS of the fish samples. Typical spectra of the analyzed fish samples from Palmar in both campaigns. Extended m/z range of the spectra shown in Figure 4. (A) Unexposed fish (LMB); (B) February 2017 sample with MCs: m/z = 995.5 and 1116.6 (which correspond to the single protonated ion of MC-LR and MC-LR-Cys); (C) March 2021 sample with MCs: m/z = 995.6, 1023.6, 1037.6, and 1068.5 (which correspond to MC-LR, [D-Leu1,Dha7] MC-LR, [D-Leu1] MC-LR, and MC-WR, respectively).

Table S1. Data of the MC results in fish by Nb-ELISA and Nb-QMALDI MS (2017 and 2021).

2017 Sample ID	Nb-ELISA—ng/g Fish	Nb-QMALDI (Total MCs—ng/g Fish)	2021 Sample ID	Nb-ELISA—ng/g Fish	Nb-QMALDI (Total MCs—ng/g fish)
1	168.65	61.25	1	167.03	89.89
2	9.47	1.62	2	275.74	184.17
3	105.53	84.02	3	304.85	191.39
4	14.71	2.76	4	79.43	50.10
5	1.18	0.42	5	81.44	48.15
6	34.18	13.43	6	59.80	35.88
7	23.12	17.04	7	11.02	4.55
8	1.18	1.43	8	43.49	19.24
9	13.71	7.31	9	19.54	12.18
10	62.18	50.53	10	43.61	6.02
11	31.18	21.10	11	1.18	0.42
12	11.71	9.74	12	25.36	11.30
13	3.71	3.78	13	20.68	10.72
14	5.88	2.12	14	11.61	6.92
15	20.18	12.13	15	12.00	3.66
16	57.76	38.20	16	4.,68	32.43
17	2.88	0.84	17	47.46	32.14
18	1.18	0.42	18	11.48	5.12
19	7.29	1.39	19	25.75	18.94
20	1.80	1.22	20	30.17	28.14
21	1.18	0.42	21	31.47	31.71
22	1.18	0.42	22	64.59	64.45
23	26.86	27.62	23	59.73	63.83
24	1.18	0.42	24	23.61	16.53
25	1.50	0.62	25	3.24	2.58
26	1.18	0.42	26	15.95	19.20
			27	42.49	32.99
			28	3.22	2.79
			29	1.18	0.42
